# Supplementary material for: Geometric Tuning of Cytokine Receptor Association Modulates Synthetic Agonist Signaling
Source: bioRxiv. 2025 Oct 13:2025.10.12.681819. Preprint. [Version 1] doi: 10.1101/2025.10.12.681819 (PMC12632830; doi:10.1101/2025.10.12.681819)
Supplement: 1 [file NIHPP2025.10.12.681819v1-supplement-1.pdf]

bioRxiv preprint doi: https://doi.org/10.1101/2025.10.12.681819; this version posted October 13, 2025. The copyright holder for this preprint (which was not certified by peer review) is the author/funder, who has granted bioRxiv a license to display the preprint in perpetuity. It is made available under aCC-BY 4.0 International license.

Supplementary Figures

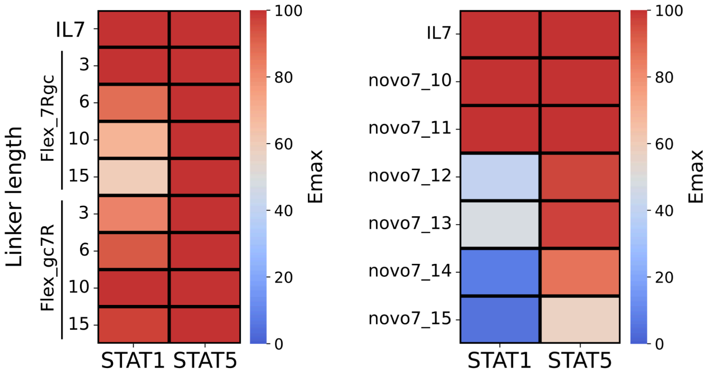

Figure S1. Reconstructing IL-7 signaling by flexibly fusing *de novo* binders.
(left), Signaling strength (Emax) for STAT1 and STAT5 for designed IL-7Rα and γc binder flexible fusions. The orientation of the fusion (IL-7Rα-γc or γc-IL-7Rα) and length in amino acids of the flexible (GGS) linker is specified for each agonist. (right), Signaling strength for designed rigid novokines.

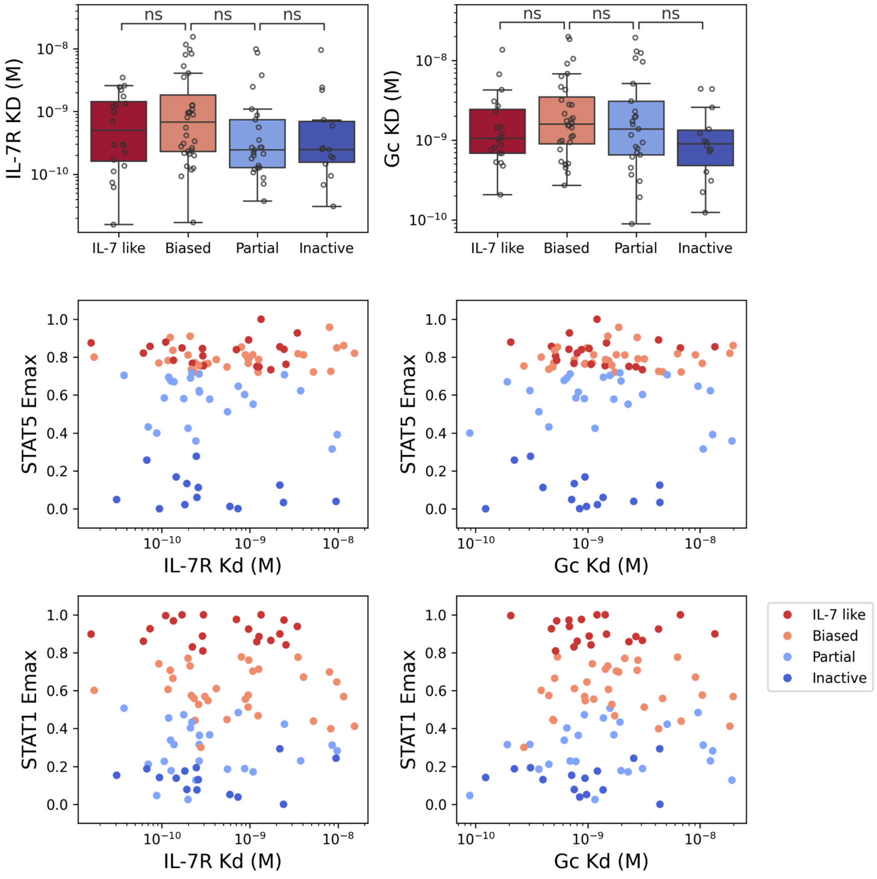

Figure S2. Relation between affinity (K_D) and signaling strength (E_{max}) for designed IL-7 novokines.
(top), Affinity of novo7 novokines grouped by their signaling pattern. n.s.= Not significant (p-value>0.05). (middle), scatterplot of affinities for each receptor and relation to pSTAT5 Emax. (bottom), scatterplot of affinities for each receptor and relation to pSTAT1 Emax.

bioRxiv preprint doi: https://doi.org/10.1101/2025.10.12.681819; this version posted October 13, 2025. The copyright holder for this preprint (which was not certified by peer review) is the author/funder, who has granted bioRxiv a license to display the preprint in perpetuity. It is made available under aCC-BY 4.0 International license.

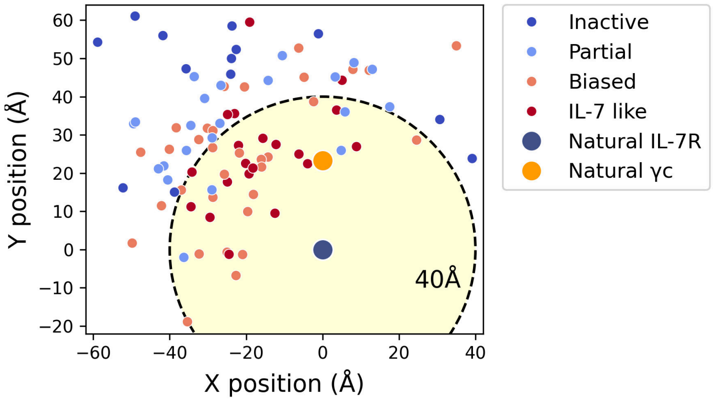

Figure S3. X-Y positions of receptor termini for novo7 novokines
Membrane-proximal receptor termini positions in a X-Y plane for designed agonists, colored by signaling pattern, all aligned using the IL-7Rα receptor as reference, and including the position of the γc in the natural IL-7 complex.

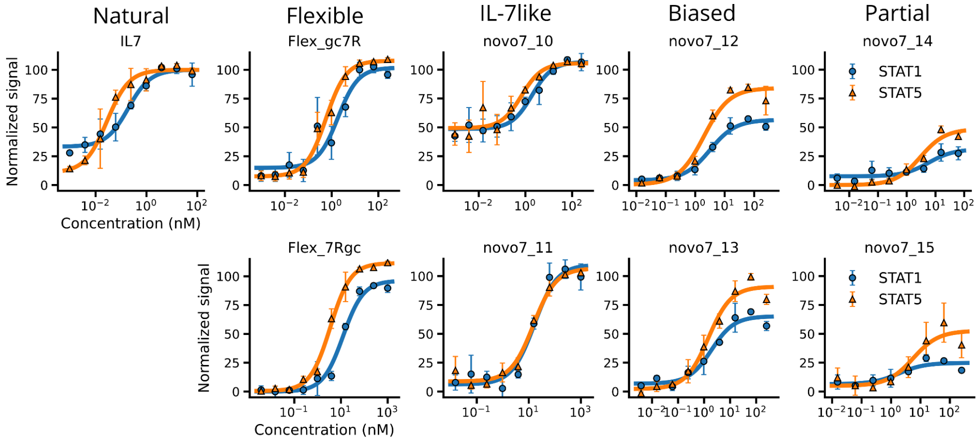

Figure S4. Dose curve for novo7 novokines analyzed by RNA-seq.
pSTAT1 (blue) and pSTAT5 (orange) signaling for novo7 agonists analyzed in RNA-seq, organized by signaling pattern, including the natural IL-7 cytokine and flexible fusions between IL-7Rα and γc binders.

bioRxiv preprint doi: https://doi.org/10.1101/2025.10.12.681819; this version posted October 13, 2025. The copyright holder for this preprint (which was not certified by peer review) is the author/funder, who has granted bioRxiv a license to display the preprint in perpetuity. It is made available under aCC-BY 4.0 International license.

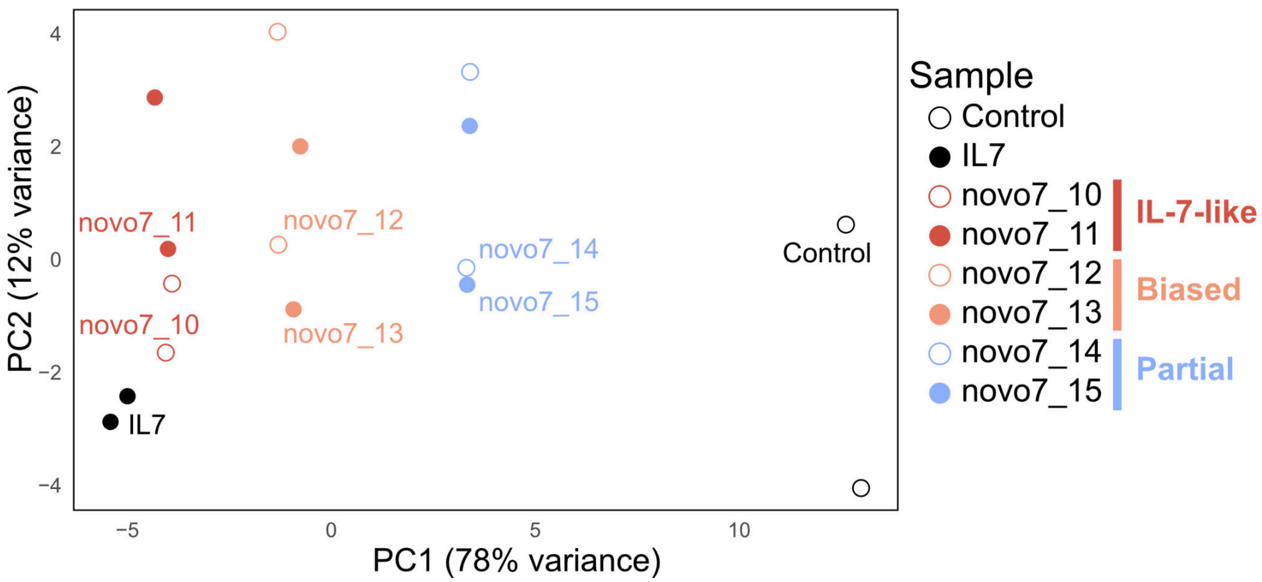

Figure S5. Principal component analysis (PCA) of RNA-seq samples colored and shaped by sample identity. Each point represents an individual RNA-seq replicate, with colors indicating functional classifications (e.g., IL-7-like, biased, partial), and point shapes distinguishing the two samples within each group. Each sample has two biological replicates. Percent variance explained by PC1 and PC2 is indicated on the axes. PC1 separates samples based on functional phenotype, while PC2, accounting for a smaller proportion of variance, captures intra-group replicate variability.

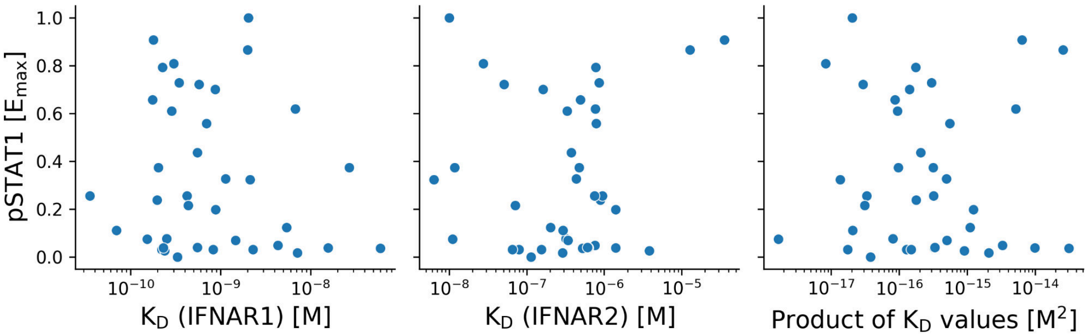

Figure S6. Relation between affinity (K_D) and signaling strength (E_{max}) for designed type I IFN novokines.

Scatterplots between pSTAT1 Emax normalized with respect to natural IFNa1, and relation to affinity measured by SPR for IFNAR1 (left), IFNAR2 (center) or the product between both (right).

bioRxiv preprint doi: https://doi.org/10.1101/2025.10.12.681819; this version posted October 13, 2025. The copyright holder for this preprint (which was not certified by peer review) is the author/funder, who has granted bioRxiv a license to display the preprint in perpetuity. It is made available under aCC-BY 4.0 International license.

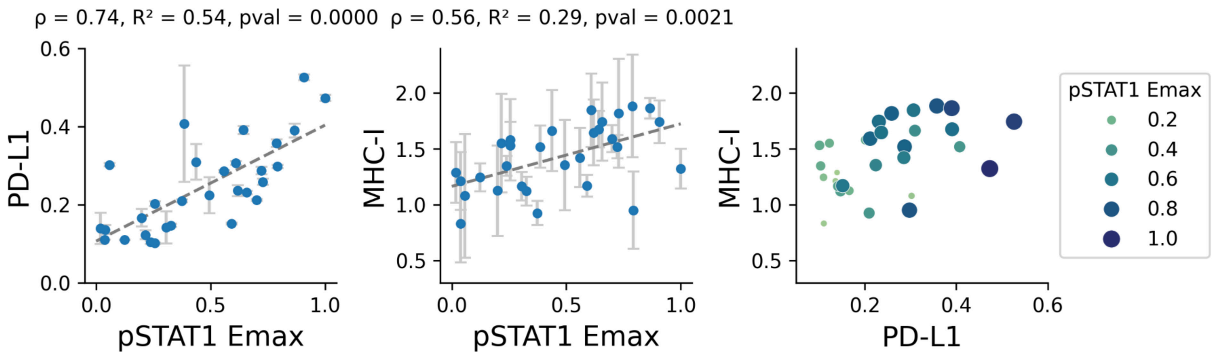

Figure S7. Scatterplots of PD-L1 and MHC-I expression in relation to pSTAT1 signaling strength. Surface expression levels of PD-L1 (left) and MHC-I (center), normalized to natural IFNα1, are plotted against the maximal pSTAT1 signaling response (Emax) elicited by each designed novoIFNα ligand. Dotted lines indicate linear regression fits, with Spearman's correlation coefficient (ρ), R^2, and permutation-derived p-values shown above each plot. The right panel displays a scatterplot of MHC-I versus PD-L1 expression, with point size reflecting the pSTAT1 Emax for each novoIFNα ligand.

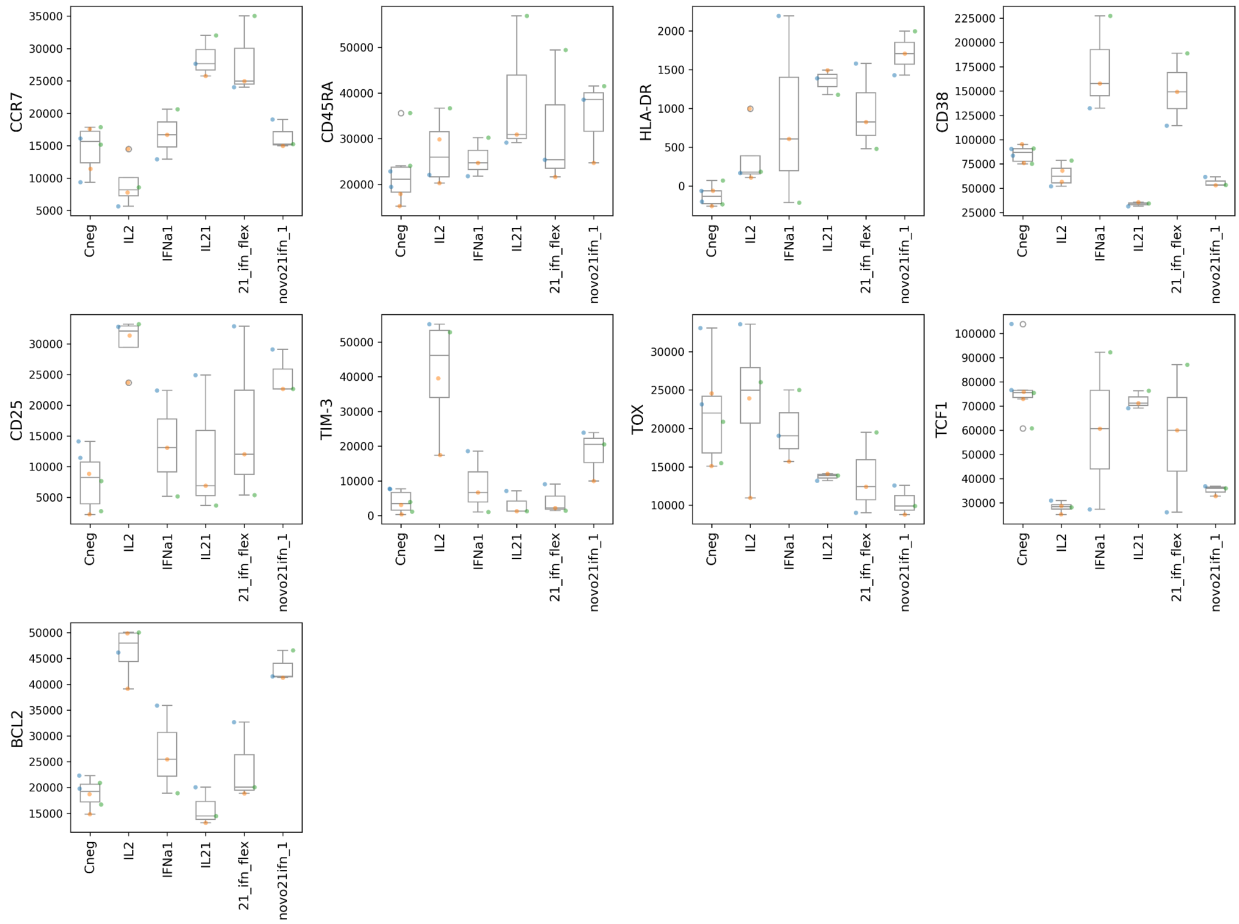

Figure S8. Box plots of selected markers in the naive CD8⁺ T cell differentiation assay. Each color indicates a different donor. Units are directly the values obtained from flow cytometry.

bioRxiv preprint doi: https://doi.org/10.1101/2025.10.12.681819; this version posted October 13, 2025. The copyright holder for this preprint (which was not certified by peer review) is the author/funder, who has granted bioRxiv a license to display the preprint in perpetuity. It is made available under aCC-BY 4.0 International license.

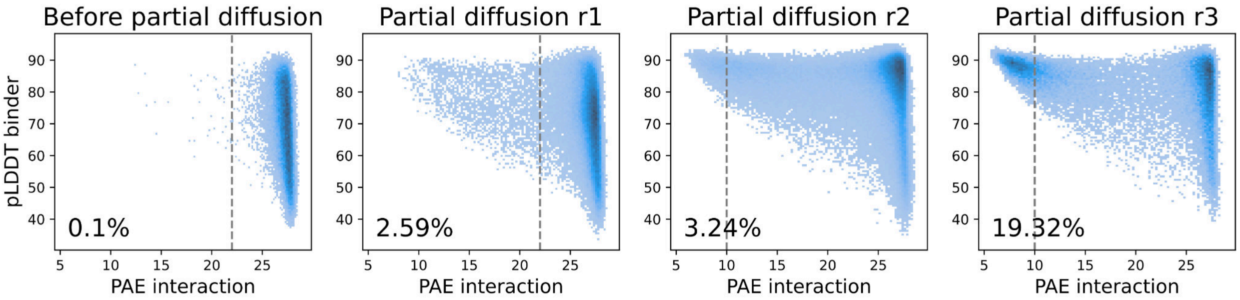

Figure S9. Improvement of binding affinity for IL-3Rα through multiple rounds of partial diffusion. Scatterplots between AlphaFold2's accuracy of prediction (pLDDT) and strength of interaction (PAE) for designed novo3 novokines across rounds of partial diffusions. The percentage of designs below a PAE threshold is shown for comparison. Final designs were below PAE<10 after the 3rd round of diffusion.
